# Herbs in exercise and sports

**DOI:** 10.1186/1880-6805-31-4

**Published:** 2012-03-08

**Authors:** Chee Keong Chen, Ayu Suzailiana Muhamad, Foong Kiew Ooi

**Affiliations:** 1 Sports Science Unit, School of Medical Sciences, Universiti Sains Malaysia, 16150 Kubang Kerian, Kelantan, Malaysia

**Keywords:** herbs, ginseng, caffeine, ephedrine, *Eurycoma longifolia *Jack

## Abstract

The use of herbs as ergogenic aids in exercise and sport is not novel. Ginseng, caffeine, ma huang (also called 'Chinese ephedra'), ephedrine and a combination of both caffeine and ephedrine are the most popular herbs used in exercise and sports. It is believed that these herbs have an ergogenic effect and thus help to improve physical performance. Numerous studies have been conducted to investigate the effects of these herbs on exercise performance. Recently, researchers have also investigated the effects of *Eurycoma longifolia *Jack on endurance cycling and running performance. These investigators have reported no significant improvement in either cycling or running endurance after supplementation with this herb. As the number of studies in this area is still small, more studies should be conducted to evaluate and substantiate the effects of this herb on sports and exercise performance. For instance, future research on any herbs should take the following factors into consideration: dosage, supplementation period and a larger sample size.

## Background

According to botanists, 'herb' is defined as a soft-stemmed plant which dies after flowering, whereas herbalists define an 'herb' as any part of a plant which can be used for medicine, cooking, cosmetics and as a scent or dye. In nature, there are many types of herbs that can be found and have been used traditionally for many purposes. Athletes are among those who use herbs for their own benefit. They believe that some herbs may help them to improve their performance, speed up recovery, maintain health and fitness during intense periods of training, increase muscle mass and reduce body fat. Ginseng, caffeine, ephedrine and recently *Eurycoma longifolia *Jack are among the popular herbs used to enhance exercise and sports performance. In this article, we review some of the studies of these herbs to investigate their effects on exercise and sports performance.

'Ginseng' is a general name for the plant genus *Panax*. Some of the members of genus are *Panax ginseng*, *Panax quinquefolius*, *Panax notoginseng *and *Panax japonicus*. Among these plants, *Panax ginseng*, also known as Chinese or Korean ginseng, is the one which is most widely used [1]. Ginseng is available in various forms, such as whole root, root powder, teas and tinctures, as well as standardized root extracts containing known amounts of ginsenosides in every batch [2]. Ginseng roots contain approximately 13 glycosylated steroidal saponins (ginsenosides) which are the probable active agents [1,3,4]. Ginseng is thought to be a tonic that can improve vitality, health and longevity. Separation of ginsenosides and administration to animals has revealed activities which stimulate the central nervous system [2-6]. Other probable functions of ginseng include increased production of corticotropin and cortisol in animals and humans and anabolic actions in animals [2-4,7-9]. In addition, ginseng has been shown to possess antioxidant properties whereby it scavenges hydroxyl radical and inhibits lipid peroxidation [10]. Ginseng has also been touted as possessing a stimulant effect and thus improves alertness and decreases fatigue and stress [11]. Consequently, various possible mechanisms of ginseng ingestion have been postulated to contribute to the enhancement of human sports performance.

In a number of animal studies, ginseng has been reported to improve exercise performance. Researchers in some other animal studies, however, have reported that the use of large doses or parenteral administration could weaken extrapolation of these scientific data to humans [3,4,12]. In exercise and sports science, ginseng is believed to be a physical performance enhancer [1]. However, a review of the available data on the effects of ginseng on human exercise performance reveals dose-response and duration effects, which may account for the equivocal results reported [13]. Its chronic use has been believed to improve cardiorespiratory function and lower lactate concentration in the blood in addition to improving physical performance [14]. Nevertheless, it has been reported that its benefits were best seen in individuals in poor physical condition [4].

Many study investigators have found that ginseng can increase exercise duration until exhaustion during forced exercise trials [4,14]. This was believed to be due to stress adaptation via ginseng supplementation [11]. *Panax ginseng *has been investigated extensively for its stress-attenuating activity [15]. It is a well-known adaptogen and has been shown to be effective in attenuating stress-induced adverse effects in astronauts and soldiers [16]. Rai and colleagues [17] demonstrated that *Panax ginseng *has potent adaptogenic activity that is mediated by regulation of pituitary adrenocorticotropic hormone secretion to combat stress. This adaptation to stress may increase exercise time to exhaustion [1]. For example, Liang *et al. *[18] reported that, in untrained adults, consumption of one 1, 350-mg *Panax notoginseng *capsule per day for 30 days improved their endurance time by more than 7 minutes and lowered their maximal mean blood pressure and maximal oxygen consumption (VO_2_max) at the 24th minute during endurance cycling exercise. McNaughton *et al. *[19] conducted a placebo-controlled cross-over study in which the subjects were given Chinese ginseng, Siberian ginseng or placebo supplements (1 g/day for 6 weeks for each supplement). They reported that Chinese ginseng significantly increased maximal oxygen consumption, postexercise recovery and pectoral and quadriceps strength, but handgrip strength did not change after the supplementation regimen. Furthermore, it has been shown that a single dose of *Panax ginseng *(200 mg) can modulate circulating blood glucose level, enhance cognitive performance on a mental arithmetic task and ameliorate the increase in subjective feeling of mental fatigue during sustained intense cognitive processing [20].

It has been reported that the use of ginseng does not lead to any positive test results for banned substances after urine testing of elite athletes, although ginsenosides and their metabolites were detectable in the sera and urine of athletes following the ingestion of ginseng [21,22]. *Panax ginseng *has certain ergogenic properties that may improve both physical and mental performance, provided that the dosage is adequate (≥ 200 mg/day) and the supplementation period is of sufficient duration (≥ 8 weeks). In addition, supplementation of *Panax ginseng *has been proven to be safe on the basis of animal toxicity studies which demonstrated that ginseng does not result in teratogenicity or mutagenicity [3,4].

Researchers in related studies have revealed that caffeine supplementation can improve performance at varying intensities and modalities of exercise [23], and evidence of its effects on submaximal exercise has also been well-documented [24]. However, its effect on intermittent sprint performance is still lacking [23]. It has been reported that plasma concentration of caffeine is maximal 1 hour after ingestion and returns to normal 6 hours after ingestion [25]. Thus, for ergogenic purposes, a dose of caffeine ranging from 2 to 9 mg/kg body mass has been suggested to be effective, and the caffeine should be taken at least 1 hour prior to exercise or competition [26].

The effect of caffeine on endurance was reported in a study where there was a significant increase (44%) in endurance running performance after athletes ingested 9 mg/kg body mass of caffeine 1 hour prior to exercise [27]. Caffeine is a well-documented stimulant of the central nervous system as well as the cardiovascular and respiratory systems. It is believed that caffeine ingestion increases blood catecholamine concentration [28,29]. Furthermore, its ergogenic effect on sports performance is also attributed to its effect on substrate availability, namely, free fatty acids [30]. Increases in free fatty acid concentration in turn lead to a glycogen-sparing effect [28] because the body's energy system will start using free fatty acid as a primary source of fuel. A recent study demonstrating endurance enhancement with caffeine supplementation (5 mg/kg body weight) showed a significantly higher level of plasma free fatty acids in the caffeine trial compared to the placebo trial [31]. Glycogen sparing can delay the onset of exhaustion; consequently, physical performance can be enhanced [32,33]. Researchers in two studies who used the needle biopsy procedure demonstrated a glycogen-sparing effect following caffeine supplementation [34,35]. In addition, Kamat *et al. *[36] proposed that the ergogenic effect of caffeine could be due to its antioxidant property. However, more studies need to be conducted to substantiate this claim.

Collomp *et al. *[37] investigated the beneficial effects of caffeine ingestion on sprint performance in trained and untrained swimmers. Subjects' swimming velocity and blood lactate concentrations were observed to determine the plausible effects of caffeine. The subjects were required to perform 100-metre freestyle swimming twice at maximal speed: once after ingestion of 250 mg of caffeine and the other after placebo ingestion. These two tests were separated by 20 minutes of passive recovery. The study results showed that the swimming velocity of the trained swimmers was significantly improved after caffeine ingestion and that maximal blood lactate concentration was significantly enhanced in both untrained and trained subjects after caffeine ingestion.

Caffeine is also associated with mental alertness and mood. Yeomans and colleagues [38] found that ingestion of 1 and 2 mg/kg caffeine at breakfast decreased reaction time and improved rated mental alertness, thus increasing performance. They also found that a 1 mg/kg dose of caffeine could increase performance accuracy. Similarly, ingestion of 1 mg/kg caffeine 60 minutes after breakfast in the subjects who did not consume any caffeine during breakfast also improved their rated mental alertness and decreased their reaction time. However, doubling caffeine ingestion did not improve those effects; the effects of caffeine in the subjects who ingested caffeine during breakfast and 60 minutes after breakfast were similar to those who ingested caffeine once, either only during breakfast or 60 minutes after breakfast. This study demonstrated that the effects of caffeine occur only in the caffeine-deprived subjects.

Chinese ephedra, or 'ma huang', is a sporophyte herb that has also been studied for its effects on exercise performance [39-43]. It is found mainly in Pakistan, China and northwestern India. The active ingredients consist of ephedrine and related alkaloids [44]. It is a sympathomimetic alkaloid because it mimics epinephrine effects and stimulates the sympathetic nervous system [45]. Receptors for ephedrine in human body are found on most cells, including the heart, lungs and blood vessels. Ephedrine was claimed to have ergogenic properties whereby it was believed to improve aerobic performance and endurance by reducing fatigue, increasing alertness, improving reaction time and increasing strength [41]. However, a number of studies in which investigators examined the effects of ephedrine or pseudoephedrine on exercise performance in humans have demonstrated that there was no enhancement of performance when the normal dosages (≥ 120 mg/day) considered safe were ingested [46-48]. Sidney and Lefcoe [46] gave 24-mg ephedrine supplements to 21 males and reported no significant differences compared with placebo in muscle strength, endurance or power, speed, lung function, hand-eye coordination, reaction time, anaerobic capacity and cardiorespiratory endurance, maximal oxygen consumption and ratings of perceived exertion. Swain *et al. *[48] administered 1 and 2 mg/kg pseudoephedrine or 0.33 and 0.66 mg/kg phenylpropanolamine or placebo to ten trained cyclists who were then required to undergo testing on a bicycle ergometer and a urine drug test. There were no significant differences in maximal oxygen consumption, ratings of perceived exertion, blood pressure, peak pulse rate or time to exhaustion between trials. Similarly, Gillies *et al. *[47] reported no significant changes in a cycling time trial performance or muscle function when ten subjects were given either 120 mg of pseudoephedrine or placebo in a randomized, double-blind, placebo-controlled cross-over study.

It is postulated that the positive effects of ephedrine can be seen when it is combined with caffeine on the basis of studies in which caffeine was believed to potentiate the effects of ephedrine [39,41]. For instance, the combination of caffeine and ephedrine has been found to increase the time to exhaustion during a standard high-intensity cycle ergometer exercise test [39]. Another study showed that after ingesting 75 mg of ephedrine and 375 mg of caffeine, there was a significant decrease in the completion of a 3.2-km run [40]. In another study, 10 km of running on a treadmill was 1 minute faster after the ingestion of ephedrine or a combination of ephedrine and caffeine when compared to the placebo trial [42]. These data raise speculation that the effect of the combination of caffeine and ephedrine occurs as a result of central nervous system stimulation. Furthermore, it has been reported that ingestion of this combination decreased the rate of perceived exertion during high-intensity exhaustive exercise [49]. Individual ephedrine alkaloids do not seem to augment physical performance, but, when combined with caffeine, they apparently have a synergistic effect that prolongs exercise time to exhaustion.

*Eurycoma longifolia *Jack is one of the herbs found in Malaysia. It is commonly known as 'tongkat ali' in Malaysia and as 'pasak bumi' in Indonesia. It is also referred to as 'Malaysian ginseng' because it is well-known among various ethnic groups in Malaysia as a treatment for various diseases and enhancing health [50]. *Eurycoma longifolia *Jack is a tall, single-stemmed, slender, shrubby, slowly growing tree, and it can be found on sandy soil. It belongs to the *Simaroubaceae *family and grows wildly in Southeast Asian countries, that is, Malaysia, Indonesia, Thailand, Myanmar, Laos and Cambodia [50-52].

This herb has been used as an anticoagulant for complications during childbirth, a treatment for dysentery [52], an aphrodisiac [53,54], an antimalarial agent [55,56], an antibacterial ointment [57,58], an anticancer medicine [58], an antihyperglycaemic therapy [59] and an anxiolytic [60]. The pharmacological activity of this plant is actually attributed to these various quassinoids and also to the squalene derivatives biphenylneolignans, tirucallane-type triterpenes, canthine-6-1 and β-carboline alkaloids [51]. Mohd Tambi [61] reported that consumption of water-soluble extract of *Eurycoma longifolia *Jack, even at a high dose of 600 mg, is nontoxic in humans.

Published scientific data regarding the effects of *Eurycoma longifolia *Jack on exercise performance are scarce. Nevertheless, the acute effects of an herbal drink containing *Eurycoma longifolia *Jack on cycling endurance performance were investigated [62,63]. In this previous study, subjects were given a low dosage of *Eurycoma longifolia *Jack (approximately 0.67 mg of *Eurycoma longifolia *Jack per trial) during endurance cycling performance. The young, trained male cyclists ingested either an herbal drink or placebo and cycled as long as possible at 70% VO_2_max for the first hour and at 80% VO_2_max thereafter until exhaustion during the experimental trials. It was reported that there was no significant improvement in cycling performance or in the physiological responses between the two trials in this study. It is speculated that the results could be due to the inadequate concentration of *Eurycoma longifolia *Jack in the drink (0.1 mg/100 ml of drink) which was given to the subjects during the cycling trials.

Subsequently, we conducted another study to investigate the effects of *Eurycoma longifolia *Jack on endurance running performance with a higher dosage of this herb and a longer supplementation duration [64]. Twelve recreational athletes were recruited to participate in this study, in which they were asked to consume two capsules of the supplement (75 mg of *Eurycoma longifolia *Jack per capsule) or placebo capsules daily for 7 days before and again 1 hour prior to the exercise trial. We observed that this amount of *Eurycoma longifolia *Jack (150 mg daily for 7 days) had no beneficial effects on the participants' endurance running performance and physiological responses. However, it has been reported that *Eurycoma longifolia *Jack supplementation (150 mg for 5 weeks) can increase muscle strength [65]. Therefore, we believe that the supplementation period and maybe the dosage used in our previous study were still insufficient to elicit the beneficial effects of *Eurycoma longifolia *Jack on endurance performance and physiological responses. Thus further study at higher dosages and for longer supplementation periods may be warranted to determine its effects on exercise and sports performance.

Some of these herbs have been shown to have beneficial effects on psychological states. For example, ginseng has positive effects on stress, caffeine improves mental alertness and mood and *Eurycoma longifolia *Jack has anxiolytic (that is, antianxiety) properties. However, how these changes in psychological states as a result of herbal supplementation affect sports performance has not been well-studied. Hence further studies could also focus on the effects of these herbs on psychological states and determine if these effects (if any) are associated with a concomitant improvement in sports performance.

## Conclusions

Table 1 summarises the selected studies on the effects of ginseng, caffeine, ephedrine, a combination of caffeine and ephedrine, and *Eurycoma longifolia *Jack in exercise and sports performance. It can be observed from the data in this table that researchers have used various types of herbs to determine their effectiveness in enhancing sports performance. To date, the findings regarding their purported ergogenic effects are still inconclusive. The reason for these equivocal findings could be due to the differences in physiological responses of each individual toward the supplementation of these herbs. For example, there could be differences in terms of absorption, transport and storage of the active ingredients in the body of the participants in the studies. Furthermore, individual differences in physical attributes such as fitness level, body composition, age and sex could have resulted in varied responses toward the types of herbs consumed. Nevertheless, our review of the available literature led us to the following conclusions.

**Table 1:** Selected studies on the effects of ginseng, caffeine, ephedrine, combination of caffeine and ephedrine, and *Eurycoma longifolia *Jack in exercise and sports

Studies	Population	Herbal treatment	Exercise	Key findings
*Panax ginseng*				
Kim et al., 2005 [14]	Seven healthy males	2 g of *Panax ginseng *extract or placebo three times per day for 8 weeks	Exhaustive incremental exercise on treadmill	Increased exercise duration until exhaustion and facilitation of recovery from exhaustive exercise
Liang et al., 2005 [18]	29 untrained adults (ages 20 to 35 years old)	For 30 days: 1, 350 mg/day *Panax ginseng *or placebo	Endurance exercise on cycle ergometer at 65% to 70% VO_2 _peak. Exercise intensity increased by 30 W at every 5-minute interval after first 35 minutes of exercise until exhaustion.	Improved endurance exercise time to exhaustion with *Panax ginseng *consumption
Caffeine				
Ping et al., 2010 [31]	Nine male recreational runners	1 hour prior to exercise: 5 mg/kg body weight of caffeine or placebo	Running to exhaustion at 70% VO_2_max on a motorised treadmill in the heat (31°C and 70% relative humidity)	Improved endurance running performance in the heat
Bell and McLellan, 2003 [24]	Nine males	1 hour before exercise: 5 or 2.5 mg/kg body mass of caffeine or placebo	Exercise rides to exhaustion on cycle ergometer at 80% VO_2_max performed in the morning and 5 hours later on the same day	Increased exercise time to exhaustion
Cohen et al., 1996 [28]	Seven endurance-trained competitive road racers (ages 23 to 51 years old)	0, 5 or 9 mg/kg body mass of caffeine	Maximal effort of 21-km road racers outdoors in hot and humid conditions	Race performance in high heat stress not affected by caffeine supplementation
Collomp et al., 1992 [37]	Seven trained and seven untrained subjects	Single dose of 250 mg of caffeine or placebo	2 × 100-metre freestyle swims at maximal speed separated by 20 minutes of passive recovery	Trained subjects exhibited significant improvement in swimming velocity after caffeine supplementation
Costill et al., 1978 [25]	Nine competitive cyclists	Ingestion of coffee containing 339 mg of caffeine or exercise without caffeine	Exercise until exhaustion on a bicycle ergometer at 80% VO_2_max	Cycling time with caffeine ingestion greater than non-caffeine fluid ingestion
Graham and Spriet, 1991 [27]	Seven trained competitive runners	1 hour before exercise: 9 mg/kg body mass of caffeine or placebo	Four exercise trials at approximately 85% VO_2_max: two trials of running to exhaustion and two trials of cycling to exhaustion.	Endurance time increased with caffeine supplementation in both exercise modes
Schneiker et al., 2006 [23]	Ten male team sport athletes	6 mg/kg body mass of caffeine or placebo 1 hour before exercise	Two 36-minute halves, with each half composed of 18 × 4-second sprints and 2 minutes of active recovery at 35% VO_2 _peak between each sprint	Total amount of sprint work performed and mean peak power score achieved during sprints were greater with caffeine ingestion in both exercise halves
Ephedrine				
Sidney and Lefcoe, 1977 [46]	21 healthy males (ages 19 to 30 years old)	A single dose of ephedrine (24 mg) or placebo	Muscle strength, endurance and power exercise	No effect on any of the measurements of physical work capacity
Caffeine + ephedrine				
Bell and Jacobs, 1999 [40]	Nine male recreational runners	2 hours before trials: combination of 375 mg of caffeine and 75 mg of ephedrine or placebo	Trials of the Canadian Forces Warrior Test (3.2-km run wearing 'fighting order' which weighed about 11 kg).	Run time significantly faster in the treatment group compared with placebo, and test performance was improved by caffeine and ephedrine
Bell et al., 2000 [49]	12 healthy untrained males	1.5 to 2 hours before exercise: 5 mg/kg body mass of caffeine plus 0.8 mg/kg body mass of ephedrine, 4 mg/kg body mass of caffeine plus 1 mg/kg body mass of ephedrine, 4 mg/kg body mass of caffeine plus 0.8 mg/kg body mass of ephedrine, or placebo	Cycling to exhaustion on a cycle ergometer at 85% VO_2 _peak	Time to exhaustion in the treatment trial greater than placebo
Bell et al., 1998 [39]	Eight males	1.5 hours before exercise: 5 mg/kg body mass of caffeine, 1 mg/kg body mass of ephedrine, combination of both caffeine and ephedrine, or placebo	Exercise on a cycle ergometer at a maximal power output until exhaustion	Only the combination of caffeine and ephedrine led to a significantly longer time to exhaustion than placebo
Bell et al., 2002 [42]	12 subjects	4 mg/kg body mass of caffeine, 0.8 mg/kg body mass of ephedrine, combination of both caffeine and ephedrine, or placebo	10-km run in a climatic suite at 12°C to 13°C on treadmill while wearing a helmet and backpack weighing 11 kg. Speed was regulated by subjects.	Running time decreased in ephedrine and combination of ephedrine and caffeine trials. Running pace increased in ephedrine compared with nonephedrine groups over the last 5 km of the run.
Williams et al., 2008 [43]	Nine resistance-trained male participants	45 minutes before exercise: 300 mg of caffeine, 300 mg of caffeine plus 60 mg of ephedrine, or 300 mg of glucose placebo	Maximal strength exercise of bench press (BP) at one repetition maximum (1 RM) and latissimus dorsi pull-down (LP) at 1 RM. Each subject also performed repeated repetitions at 80% of 1 RM on both BP and LP until exhaustion.	Increased alertness and enhanced mood after supplementation of combination of caffeine and ephedrine. No differences in muscle strength, endurance or peak aerobic power.
*Eurycoma longifolia *Jack				
Muhamad et al., 2010 [64]	12 recreational male athletes (age 23.3 ± 3.7 years old SD)	Two capsules per day containing 75 mg of *Eurycoma longifolia *Jack or placebo for 7 days before and another two capsules 1 hour before exercise trial	60-minute run on treadmill at 60% VO_2_max followed by 20-minute time trial	Running distance during time trial with *Eurycoma longifolia *Jack was not different from placebo. Physiological responses were also not different between trials.
Ooi et al., 2001 [62], 2003 [63]	Six male cyclists	Ingestion of herbal drink containing 0.1 mg of *Eurycoma longifolia *Jack per 100 ml of drink (about 0.67 mg of *Eurycoma *per trial) or placebo drink during exercise	Cycling at 70% VO_2_max for the first hour and 80% VO_2_max thereafter until exhaustion	No significant improvement in cycling performance or physiological responses

VO_2_max, maximal oxygen consumption; VO_2 _peak, peak oxygen consumption.

*Panax ginseng*, when administered at an adequate dosage (between 200 and 400 g/day) for a period of longer than 8 weeks may improve physical performance.

Caffeine consumption 1 hour prior to exercise at a prescribed dosage of 2 to 9 mg/kg body mass may prolong exercise time to exhaustion. Ma huang did not exert any ergogenic effect on sports performance when it was taken alone, but there is some evidence of improved physical performance when it is combined with caffeine, .

*Eurycoma longifolia *Jack, or 'tongkat ali', has not appeared to elicit any ergogenic effect on endurance performance in a limited number of studies of these herbs. However, future studies of this herb are definitely warranted because there might be a dose-dependent response and the supplementation duration of the previous studies might have been too short.

## Competing interests

The authors declare that they have no competing interests.

## Authors' contributions

All authors participated in the design of the study of *Eurycoma longifolia *Jack. AS and FK searched for more articles and journals related to the effects of supplements on exercise and sports performance. All authors read and selected relevant information and data from these articles and journals that were included in the text of this review. AS prepared the first draft of this review. FK and CK revised the draft manuscript that was submitted to the editorial board of this journal. CK made the necessary amendments that were recommended by the reviewers. All authors read and approved the final manuscript.
